# Overexpression of Chitinase 3-Like 1/YKL-40 in Lung-Specific IL-18-Transgenic Mice, Smokers and COPD

**DOI:** 10.1371/journal.pone.0024177

**Published:** 2011-09-07

**Authors:** Yuki Sakazaki, Tomoaki Hoshino, Satoko Takei, Masanori Sawada, Hanako Oda, Shin-ichi Takenaka, Haruki Imaoka, Kazuko Matsunaga, Toshio Ota, Yuzuru Abe, Ichiro Miki, Kiminori Fujimoto, Tomotaka Kawayama, Seiya Kato, Hisamichi Aizawa

**Affiliations:** 1 Division of Respirology, Neurology, and Rheumatology, Department of Medicine, Kurume University School of Medicine, Kurume, Fukuoka, Japan; 2 Drug Discovery Research Laboratories, Kyowa Hakko Kirin Co., Ltd., Shizuoka, Japan; 3 Department of Radiology and Center for Diagnostic Imaging, Department of Medicine, Kurume University School of Medicine, Kurume, Fukuoka, Japan; 4 Division of Pathology and Cell Biology, Graduate School and Faculty of Medicine, University of the Ryukyus, Nishihara, Okinawa, Japan; Ludwig-Maximilians-Universität München, Germany

## Abstract

We analyzed the lung mRNA expression profiles of a murine model of COPD developed using a lung-specific IL-18-transgenic mouse. In this transgenic mouse, the expression of 608 genes was found to vary more than 2-fold in comparison with control WT mice, and was clustered into 4 groups. The expression of 140 genes was constitutively increased at all ages, 215 genes increased gradually with aging, 171 genes decreased gradually with aging, and 82 genes decreased temporarily at 9 weeks of age. Interestingly, the levels of mRNA for the chitinase-related genes chitinase 3-like 1 (Chi3l1), Chi3l3, and acidic mammalian chitinase (AMCase) were significantly higher in the lungs of transgenic mice than in control mice. The level of Chi3l1 protein increased significantly with aging in the lungs and sera of IL-18 transgenic, but not WT mice. Previous studies have suggested Chi3l3 and AMCase are IL-13-driven chitinase-like proteins. However, IL-13 gene deletion did not reduce the level of Chi3l1 protein in the lungs of IL-18 transgenic mice. Based on our murine model gene expression data, we analyzed the protein level of YKL-40, the human homolog of Chi3l1, in sera of smokers and COPD patients. Sixteen COPD patients had undergone high resolution computed tomography (HRCT) examination. Emphysema was assessed by using a density mask with a cutoff of −950 Hounsfield units to calculate the low-attenuation area percentage (LAA%). We observed significantly higher serum levels in samples from 28 smokers and 45 COPD patients compared to 30 non-smokers. In COPD patients, there was a significant negative correlation between serum level of YKL-40 and %FEV_1_. Moreover, there was a significant positive correlation between the serum levels of YKL-40 and LAA% in COPD patients. Thus our results suggest that chitinase-related genes may play an important role in establishing pulmonary inflammation and emphysematous changes in smokers and COPD patients.

## Introduction

Chronic obstructive pulmonary disease (COPD) is an important pulmonary inflammatory disease whose prevalence and associated mortality rates have been increasing [1], [2]. In this disease, T cells (predominantly IFN-γ-producing CD8^+^ T cells (type 1 cytotoxic T cells) and Th1 cells), neutrophils and macrophages are activated in the lungs [3], [4], producing proteases such as neutrophil elastase and matrix metalloproteinase (MMP)-9, resulting in alveolar wall destruction (emphysematous change) and mucus hypersecretion. COPD patients also show increased concentrations of MMP-1 (collagenase) and MMP-9 (gelatinase B) in bronchial lavage fluid (BALF), and higher expression of these enzymes in lung macrophages [5]. In addition, various cytokines (e.g. IL-1β, IL-6, TNF-α, IFN-γ), growth factors (e.g. EGF, GMC-SF, TGF-β), and chemokines (e.g. CCL2, CXCL1, CXCL8, CXCL9, CXCL10, CXCL11) may be involved in the development of pulmonary inflammation, emphysema, and fibrosis around small airways in COPD [6]. Furthermore, Th17 cells can also activate neutrophils, and are thought to contribute to the development of COPD [4], [6].

The proinflammatory cytokines IL-1, IL-18, and IL-33 belongs to the IL-1 family [7]. IL-18 is well known to play an important role in Th1 polarization, and can also act as a co-factor for Th2 cell development and IgE production [8]–[11]. Recently, IL-18 was reported to take part in the differentiation of Th17 cells by amplifying IL-17 production by polarized Th17 cells in synergy with IL-23 [12]. IL-18 plays important roles in the pathogenesis of inflammatory diseases such as atopic dermatitis [13], rheumatoid arthritis (RA), adult-onset Still's disease, Sjögren's syndrome, and inflammatory bowel diseases including Crohn's disease [see review [8]]. IL-18 is also involved in the development of inflammatory lung diseases including pulmonary inflammation, asthma, lung injury and idiopathic pulmonary fibrosis (IPF) [14], [15] [16]. Previously, we showed that constitutive overproduction of mature IL-18 protein in the lungs of transgenic mice resulted in severe emphysema accompanied by pulmonary inflammation [17]. A significant negative correlation between the serum IL-18 level and %FEV_1_ has also been reported in COPD [18]. Taken together, these results provide strong support for the involvement of IL-18 in the pathogenesis of COPD.

Mammals are not able to synthesize or metabolize chitin. However a number of chitinolytic chitinase-like proteins including acidic mammalian chitinase (AMCase), chitinase 3-like 1 (Chi3l1), and chitin-binding protein, belonging to the 18 glycosyl-hydrolase family, have been discovered in mice [19]. Chi3l1, which is also known as breast regression protein (BRP)-39 and cartilage gp39, and its human homolog YKL-40 (also known as human cartilage gp39), have been regarded as prototype chitinase-like proteins in mammals [19]. Recent studies have demonstrated increased levels of YKL-40 protein and/or mRNA in serum or tissues of patients with inflammatory diseases, including RA, osteoarthritis (OA), sarcoidosis, and several types of malignancy [see review [20]]. YKL-40 is thought to be a useful prognostic or diagnostic biomarker for coronary artery disease and cancer [21] [22]. In addition, YKL-40 and chitinase-like protein may be involved in the pathogenesis of asthma in humans, as well as in a mouse asthma model [23]–[25]. Recently, elevated levels of YKL-40 in serum, BALF, and/or lung tissues of COPD patients have been reported [26] [27]. In the present study, we determined mRNA expression profiles in the lungs of our murine model of COPD, the IL-18-transgenic mouse [17], using microarray analysis. We found that the levels of mRNAs for chitinase-like proteins Chi3l1, Chi3l3, and AMCase were significantly increased in the lungs of IL-18-transgenic mice as compared with control wild-type mice. Moreover, the protein levels of YKL-40 were significantly higher in serum samples from smokers and COPD patients than in those from non-smokers. In COPD patients, there was a significant negative correlation between the serum level of YKL-40 and %FEV_1_. In contrast, there was a significant positive correlation between the serum level of YKL-40 and the low-attenuation area percentage (LAA%) in COPD patients. In the light of the findings presented here, we discuss the potential roles of YKL-40 and chitinase-like protein in pulmonary inflammation and emphysema.

## Methods

### Lung-specific IL-18-transgenic (Tg) mice

We used female C57BL/6N (B6) background SPC-IL-18 Tg mice in which the mature mouse IL-18 was overproduced in the lungs under the control of the human surfactant protein (SP) C promoter [17]. We established B6 IL-13 (−/−) SPC-IL-18 Tg mice by backcrossing SPC-IL-18 Tg mouse line A with B6 IL-13 (−/−) mice, as reported previously [28]. Age-matched female B6 wild-type (WT) mice, purchased from Charles River Japan (Yokohama, Japan), were used as controls. All procedures were approved by the Committee on the Ethics of Animal Experiments, Kurume University (Approval No. H22-079-084). Animal care was provided in accordance with the procedures outlined in the “Principle of laboratory animal care” (National Institutes of Health Publication No.86-23, revised 1985).

### RNA isolation

Lung tissues were obtained from both Tg and control WT B6 mice, and frozen immediately in liquid nitrogen. Total RNA was isolated by homogenization of the lung tissues in Trizol reagent (Invitrogen, Tokyo, Japan) using a Polytron PT2100 (Kinematica AG, Littau, Switzerland), as reported previously [14].

### DNA microarray analysis

A Whole Mouse Genome Oligo Microarray Kit ® (catalog no. G4121A, Agilent Technologies, Tokyo, Japan) was used for the DNA microarray analysis. We individually hybridized 500 ng of total RNA isolated from each Tg and control mouse. RNAs isolated from WT and Tg mice were labeled with Cy5 and Cy3, respectively, and the Cy3/Cy5 ratio was used as an absolute indicator of the fold-change of expression in the SPC-IL-18 Tg mouse relative to the WT control. GeneSpring software® (Agilent Technologies) was used for analysis.

### Quantitative real-time reverse transcriptase (RT) PCR

TaqMan RT-PCR was performed using a LightCycler 480 and Universal Probe Library Probes (Roche Diagnostics, Tokyo, Japan), as reported previously [13]. Briefly, first-strand cDNAs were synthesized using oligo (dT)_12–18_ primers from total RNA (4 µg) using a First-Strand cDNA Synthesis kit (Invitrogen). TaqMan PCR reactions were carried out using premade kits from Roche Diagnostics, and 5 µl of 5-fold-diluted cDNA was used in each 20-µl reaction volume. The mixture solution was denatured for 10 min at 95°C and then subjected to 45 two-step amplification cycles, each comprising an annealing/extension step at 60°C for 25 s, followed by denaturation at 95°C for 10 s. The primer sequences were:

Mouse IL-18 sense primer: 5′- CATGTACAAAGACAGTGAAGTAAGAGG –3′;
Mouse IL-18 antisense primer: 5′-TTTCAGGTGGATCCATTTCC-3′;
Mouse Chi3l1 sense primer: 5′-AGGCTTTGCGGTCCTGAT-3′;
Mouse Chi3l1 antisense primer: 5′-CCAGCTGGTGAAGTAGCAGA-3′;
Mouse Chi3l3 sense primer: 5′-GAACACTGAGCTAAAAACTCTCCTG -3′;
Mouse Chi3l3 antisense primer: 5′-GAGACCATGGCACTGAACG-3′;
Mouse GAPDH sense primer: 5′-TGTCCGTCGTGGATCTGAC-3′;
Mouse GAPDH antisense primer: 5′-CCTGCTTCACCACCTTCTTG -3′;

Relative expression levels were determined using the delta delta Ct method. Amplification of the gene for mGAPDH was performed on all samples tested to control for variations in RNA content, and all transcript values were normalized with reference to the GAPDH mRNA level.

### ELISA assays

The whole lung tissues were homogenized in 2 ml of lysis buffer (1% Triton X-100, 10 mM Tris-HCl, 5 mM EDTA, pH 7.6) containing a protease inhibitor cocktail (Complete™ Mini, Boehringer Mannheim GmbH, Mannheim, Germany) and centrifuged at 20,000× *g* for 15 min. The supernatant was then collected and stored at −80°C until ELISA assay, as reported previously [15]. Sandwich ELISA kits were used for mouse chitinase 3-like 1 (Chi3l1) (R&D Systems, Minneapolis, MN) and YKL-40 (Quidel Corporation, San Diego, CA). The limit of kit sensitivity was 16.0 pg/mL and 20 ng/mL, respectively.

### Human subjects

Serum samples were obtained from 30 non-smokers, 28 smokers, and 45 COPD patients. Forty-five COPD patients diagnosed with COPD were monitored at Kurume University Hospital (Kurume Japan), Fukuoka University Hospital (Fukuoka, Japan), Chikugogawa Onsen Hospital (Ukiha, Japan), Kirigaoka Tsuda Hospital (Kitakyushu, Japan), Shigemoto Hospital (Shimonoseki, Japan), Keisinkai Hospital (Tosu, Japan), Tokunaga-Naika Clinic (Fukuoka, Japan), the Social Insurance Futase Hospital (Iizuka, Japan), and Arao Central Hospital (Arao, Japan). Table 1 give details (number, age, sex, GOLD Stage, smoking history, body mass index [BMI], pulmonary function, and treatment) of all subjects. All patients with COPD were diagnosed on the basis of clinical history, physical examination, chest X-ray, chest computed tomography, and pulmonary function tests in accordance with the Global Initiative for Chronic Obstructive Lung Disease (GOLD) clinical criteria for the diagnosis and severity of COPD [29]. Written informed consent was obtained from each subject. Sample collection and all procedures were approved by the ethics committee of Kurume University (Approval No. 08067).

**Table 1:** Clinical characteristics of COPD patients, smokers, and non-smokers examined in this study.

	Non-smoker	Smoker	COPD
No. of patients	30	28	45
Age (years)	64.6±2.6	61.8±2.5	67.9±1.3
Sex			
Male	13	23	38
Female	17	5	7
GOLD			
Stage I	0	0	11
Stage II	0	0	15
Stage III	0	0	11
Stage IV	0	0	8
Smoking history			
Current	0	28	23
Ex-smoker(Years since quitting smoking)	0	0	22(6.75±1.2)
Pack-years	0	35.0±3.7^*^	56.5±3.7^*^ † #
BMI	20.1±0.7	20.9±0.8	21.2±0.5
%FVC	93.97±4.8	97.0±4.4	84.9±4.1
%FEV_1_	115.8±4.4	102.9±3.5	58.5±4.0^*^ †
FEV_1_% (FEV_1_/FVC)	83.9±2.0	78.8±1.5	48.1±2.2^*^ †
Treatment			
Systemic steroids	0	0	0
LAMA	0	0	15/39 (38.5%)
ICS	0	0	12/39 (30.8%)
LABA	0	0	18/39 (46.2%)
Other bronchodilators^a^	0	0	10/39 (25.6%)
No drug treatment	30/30 (100%)	28/28 (100%)	16/39 (41.0%)

*P<0.05 vs. non-smoker.

†*P*<0.05 vs. smoker.

#Smoking history in patients with stage I , II, III, and IV COPD were 51.8±7.7, 60.8±7.9, 56.2±5.7 and 56.3±7.0 pack-years, respectively.

a : Methylxanthines or Oxitropium bromide.

COPD: Chronic Obstructive Pulmonary Disease.

GOLD: Global Initiative for Chronic Obstructive Lung Disease.

BMI: body mass index.

FVC: forced vital capacity.

FEV_1_: forced expiratory volume in one second.

LAMA: long-acting muscarinic antagonists.

ICS: inhaled corticosteroids.

LABA: long-acting β_2_-agonists.

### Pulmonary function tests

Predicted normal values for Japanese individuals were used to calculate the predicted FEV_1_, which met the Japanese Pulmonary Function Standard stipulated by the Japanese Respiratory Society [30], [31].

### Assessment of HRCT

HRCT was performed at suspended full inspiration, 1-mm slice thickness, a 10-mm gap, and the smallest field of view that include both lungs using a CT scanner (Light Speed Ultra, GE Healthcare, Milwaukee, WI, USA). The three images were obtained at three anatomical levels: (a) near the superior margin of the aortic arch (level of the upper lung zone); (b) at the level of the tracheal carina (level of the middle lung zone); and (c) at the level of the orifice of the inferior pulmonary veins (level of the lower lung zone). The lungs were divided into six zones (upper, middle, and lower on both sides), and each zone was evaluated separately. Emphysema was assessed by using a density mask with a cutoff of −950 Hounsfield units to calculate LAA% as previously reported [32] [33].

### Statistical analysis

Data were presented as the mean ± standard error of the mean (SEM). Differences between two groups were analyzed by the Wilcoxon rank-sum test. Statistical analysis was performed with the JMP 7.0.1 software package (SAS Institute Japan, Tokyo, Japan). Differences were considered significant at P<0.05.

## Results

### K-means clustering analysis of 608 genes

Lung tissues were obtained, and total RNA extracted from SPC-IL-18 Tg and control Tg negative littermate mice at 5, 9, and 13 weeks of age (n = 3 per group). It was found that the mRNA levels of 608 genes were altered <0.5-fold or >2-fold at 5, 9, and/or 13 weeks of age in the lungs of Tg mice relative to those in control Tg negative mice. We classified these 608 genes into 4 groups using K-means clustering analysis. Group 1 included 140 genes whose expression levels were constitutively enhanced in the lungs of Tg mice at 5, 9, and 13 weeks. Group 2 included 215 genes whose expression levels were elevated gradually with aging. Group 3 included 171 genes whose expression levels were decreased gradually with aging. Group 4 included 82 genes whose expression levels were decreased at 9 weeks of age. In group 1 (Table S1), we demonstrate that several genes including those for IL-18 (transgene), chloride channel calcium activated 3 (Clca3), and the chitinase-related genes chitinase 3-like 3 (Chi3l3), Chi3-like 1 (Chi3l1) and acidic mammalian chitinase (AMCase), were strongly upregulated in the lungs of Tg mice from 5 to 13 weeks of age. Immunoglobulin (Ig), Ig receptor, small inducible cytokines, complement genes, major histocompatibility (MHC) class I and class II antigens, amyloid, chemokines, and apoptosis-related genes were also constitutively increased at 5 to 13 weeks of age. In group 2 (Table S2), Igs, cholesterol 25-hydroxylase (Ch25h), small inducible cytokines, arginase, a lysosome enzyme (cathepsin), and apoptosis-related genes including caspase showed a gradual increase of expression with age. In group 3 (Table S3), several genes including spermine binding protein, cytochrome P450 (CYP4B1, CYP2F2, CYP2A4, CYP2S1), antioxidant genes (e.g. glutathione s-transferase (GST) and peroxiredoxin), G-protein-coupled receptors (GPCRs, olfactory receptor-like), aquaporin and myosin showed a gradual decrease of expression with age. Among the 82 genes of group 4, those such as GPCR (olfactory receptor-like) and P450 (26A2) showed a transient decrease at 9 weeks of age (Table S4).

### Increased levels of Chi3l1 and Chi3l3 expression in the lungs of COPD model mice

**Figure 1:**
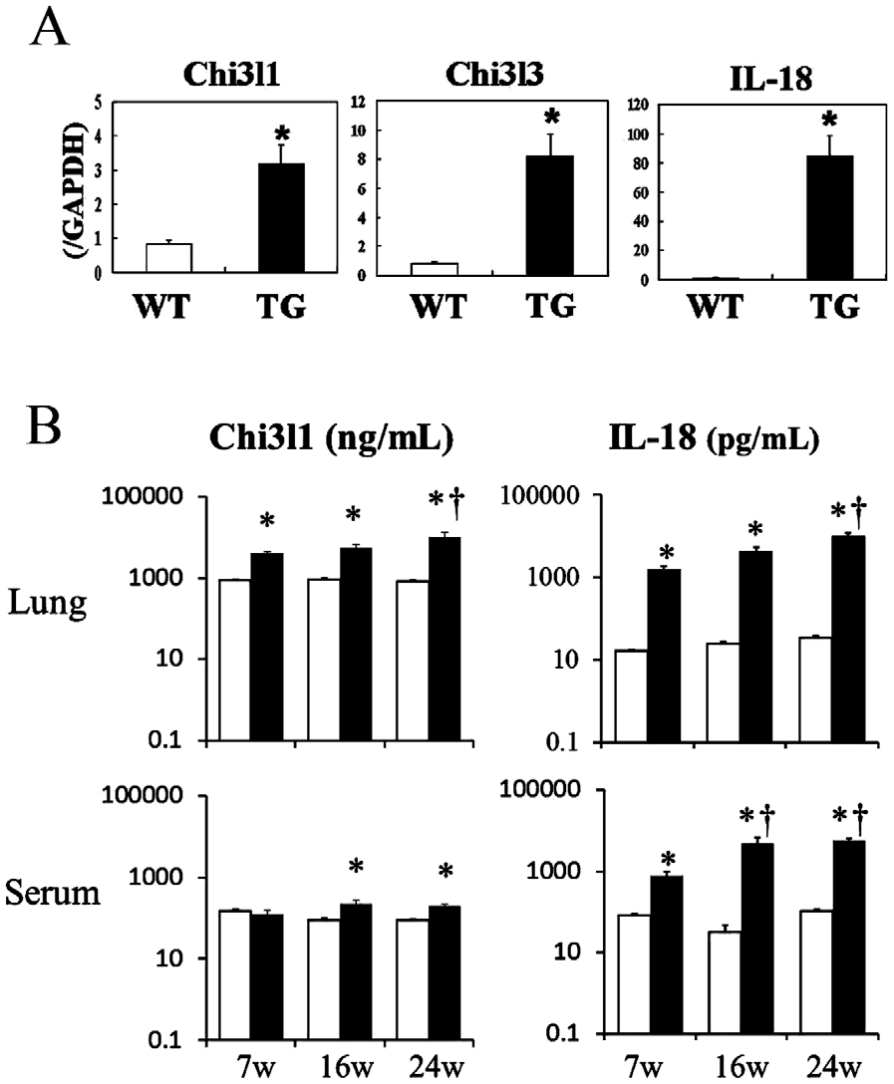
Expression of chitinase-related genes in the lungs of ling-specific IL-18 transgenic mice. (A) TaqMan RT-PCR analysis of chitinase 3-like 1 (Chi3l1), chitinase 3-like 3 (Chi3l3), and IL-18 (transgene) mRNA expression in the lungs of 5-week-old SPC-IL-18-Tg (TG) mice, in comparison with control wild type (WT) mice (n = 4, each group). ^*^ P<0.01 vs. WT mice. (B) Lung tissues and sera were obtained from 7-, 16- and 24-week-old SPC-IL-18-Tg and control WT mice (n = 4 per group). The whole lung tissues were homogenized in 2 ml of lysis buffer, as described in Methods. The levels of Chi3l1 and IL-18 protein in the lungs and sera were analyzed using sandwich ELISA kits *: P<0.01 vs. WT mice. +: P<0.05 vs. 7-week-old Tg mice.

Three chitinase-related genes, Chi3l1, Chi3l3 and AMCase, were strongly upregulated in the lungs of SPC-IL-18 Tg mice from 5 to 13 weeks (Table S1). To validate these results, TaqMan RT-PCR was used to assess RNA samples isolated from the lungs of 5-week-old female Tg and control WT B6 mice (n = 4 per each group). Quantitative RT-PCR analysis demonstrated that the expression levels of Chi3l1, Chi3l3, and IL-18 mRNAs in Tg mice were 3.7-, 10.6-, and 99.0-fold higher than in control WT mice, respectively. There were significant (p<0.01) differences in the expression levels of Chi3l1, Chi3l3 and IL-18 (transgene) in the lungs when compared to those in Tg and WT mice (Fig. 1A). Gene array analysis showed that the expressions levels of IL-18 mRNA in Tg mice were approximately 5- to 7-fold higher than in WT mice at 5, 9, and 13 weeks of age (average increase, 5.93-fold; see Table S1), similar to data that we have previously reported [17]. We then examined Chi3l1 protein levels in the lungs and sera of Tg and WT mice. Lung tissues were obtained from 7-, 16- and 24-week-old SPC-IL-18 Tg and WT control mice (n = 4 per each group) and ELISA analysis showed that the level of Chi3l1 protein in the lungs of WT mice did not alter significantly during aging, whereas there was a significant increase of Chi3l1 protein in the lungs of SPC-IL-18 Tg mice. Moreover, the level of Chi3l1 protein increased significantly (P<0.05, 7 weeks vs. 24 weeks) with age in the lungs of SPC-IL-18 Tg mice. The serum levels of Chi3l1 level were significantly increased in IL-18 Tg mice when compared with control wild type mice at 16 and 24 weeks (Fig. 1B). We also examined the levels of IL-18 protein in the lung and sera of IL-18 Tg mice. The ELISA analysis showed the levels of IL-18 in the lungs and sera were significantly increased in IL-18 Tg mice when compared with control wild type mice. The level of IL-18 protein increased significantly with age in the lungs and sera of IL-18 Tg mice (Fig. 1B), as we previously reported [17].

### IL-13 gene deletion does not reduce the level of Chi3l1 protein in the lungs of IL-18-transgenic mice

**Figure 2:**
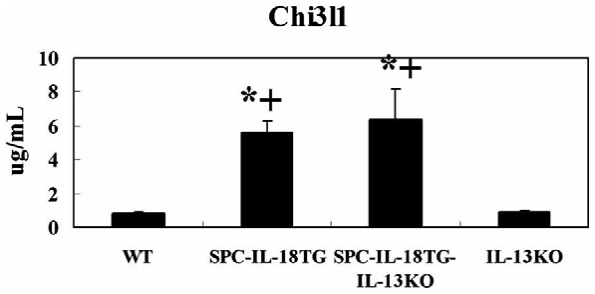
The effects of IL-13 gene deletion on the expression of Chi3l1 in the lungs of lung specific IL-18 transgenic mice. Lung tissues were obtained from 21- to 24-week-old B6 SPC-IL-18 Tg, B6 IL-13 (−/−) (KO) SPC-IL-18 Tg, B6 IL-13 (−/−) and control B6 WT mice (n = 4 per group). The whole lung tissues were homogenized in 2 ml of lysis buffer, and then the levels of Chi3l1 protein in the lungs were analyzed using sandwich ELISA kits, as described in Methods. *: P<0.05 vs. WT mice. +: P<0.05 vs. IL-13 (−/−) mice.

Previous studies have suggested that Chi3l3 and AMCase are IL-13-driven chitinase-like proteins [25], [34]. Therefore, we evaluated whether IL-13 gene deletion would reduce the level of Chi3l1 protein in the lungs of IL-18-transgenic mice. We established B6 IL-13 (−/−) SPC-IL-18 Tg mice by backcrossing SPC-IL-18 Tg mouse line A with B6 IL-13 (−/−) mice. ELISA analysis revealed that IL-13 gene deletion did not significantly reduce the level of Chi3l1 protein in the lungs of the IL-18-transgenic mice (Fig. 2).

### Expression levels of YKL-40 in sera of non-smokers, smokers and COPD patients

**Figure 3:**
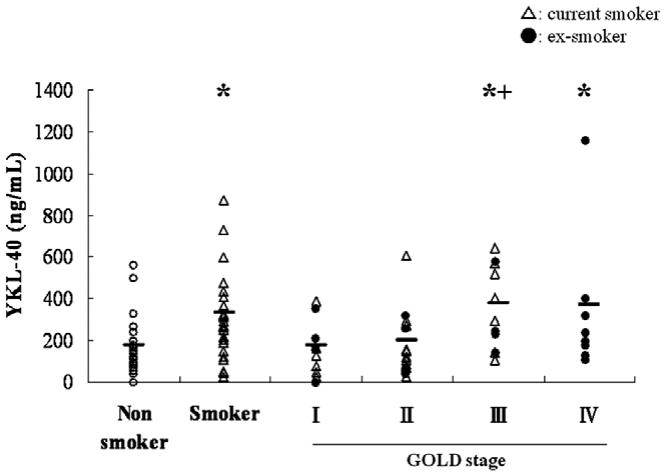
Serum levels of YKL-40 in smokers and a subset of COPD patients. Serum samples were obtained from 30 non-smokers, 28 current smokers, and 45 COPD patients (GOLD stages I [n = 11], II [n = 15], III [n = 11], and IV [8]), and analyzed for YKL-40 protein levels using sandwich ELISA kits. *: P<0.01 vs. nonsmokers. +: P<0.05 vs. GOLD stages I or II.

**Figure 4:**
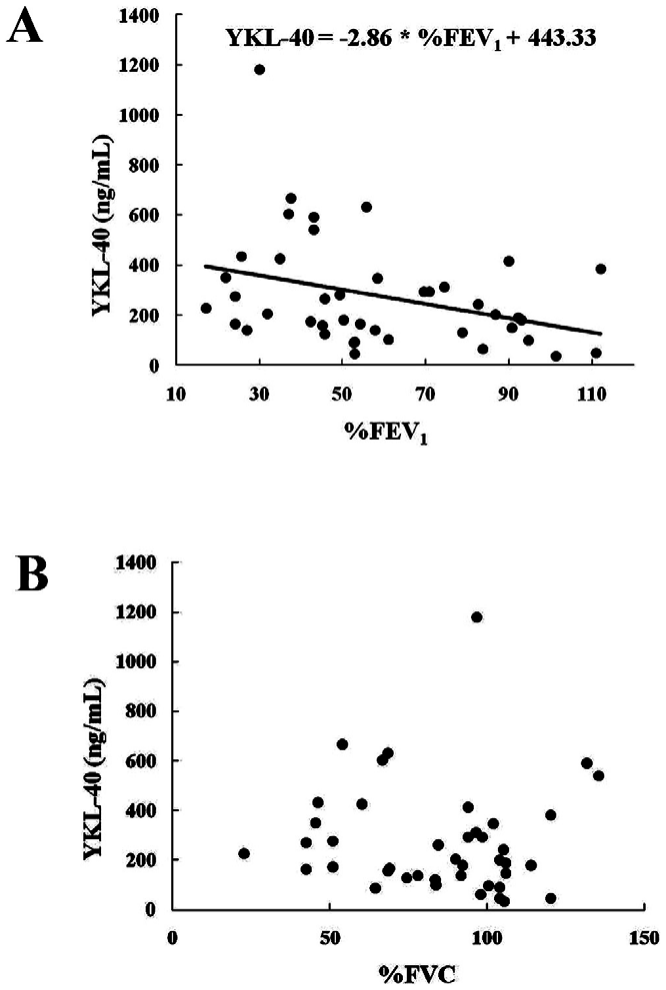
Relationship of serum levels of YKL-40 with %FEV_1_ and %FVC in COPD patients. Serum YKL-40 levels and %FEV_1_ (A) and %FVC (B) in COPD patients (n = 45) were analyzed.

Based on our data with the transgenic mouse model, we next asked if similar changes in the chitinase-like genes could also be observed in patients with significant lung inflammation, i.e. smokers and COPD patients. Data classifying the patients is shown in Table 1. Serum levels of YKL-40 in current smokers without COPD (331.8±37.0 ng/mL, n = 28) and COPD patients (268.9±32.3 ng/mL, n = 45) were significantly (P<0.01) higher than those in non-smokers (177.8±22.6 ng/mL, n = 30). The serum levels of YKL-40 did not differ significantly between 28 current smokers without COPD and the 45 COPD patients. Serum levels of YKL-40 in 23 current smokers with COPD and 22 ex-smokers with COPD were 254.5±41.1 and 283.9±51.0 ng/mL, respectively. There were no significant differences in serum YKL-40 levels between current smokers with COPD and ex-smokers with COPD. The serum levels of YKL-40 in 23 current smokers with COPD were not significantly higher than those in 28 current smokers without COPD, or in 30 non-smokers. Next, we classified the 45 COPD patients according to the GOLD classification of COPD severity . Serum YKL-40 levels in GOLD stages I (n = 11), II (n = 15), III (n = 11), and IV (n = 8) were 181.6±38.2, 199.4±39.8, 376.2±59.6, and 371.6±120.6 ng/mL, respectively. Interestingly, the serum levels of YKL-40 in GOLD stages III and IV, but not stages I, or II, were significantly (P<0.01) higher than those in non-smokers. The serum levels of YKL-40 in GOLD stage III were significantly (P<0.01) higher than those in stages I or II. It is of note that the serum levels of YKL-40 in some smokers were over 600 ng/mL (Fig. 3). Next, the correlation between serum levels of YKL-40 and pulmonary function was analyzed in nonsmokers, smokers and COPD patients. In COPD patients, there was a significant (P<0.05) negative correlation between serum level of YKL-40 and predicted FEV_1_ (%FEV_1_) (r = 0.338) (Fig. 4A) but not between serum YKL-40 and %FVC (Fig. 4B). In contrast, no significant correlations between serum levels of YKL-40 and %FEV_1_ were observed in nonsmokers or smokers (data not shown). Serum levels of IL-18 in smokers (n = 28, 216.5±15.5 pg/mL) and COPD patients (n = 40, 235.4±13.2 pg/mL) were significantly (P<0.0001) higher than those in nonsmokers (n = 30, 113.7±13.5 pg/mL), as we previously reported [18]. There was a significant association between serum levels of IL-18 and YKL-40 among nonsmokers, smokers and COPD patients (data not shown). We evaluated whether ICS treatment influenced serum levels of YKL-40 in COPD patients. However, we observed that ICS treatment did not significantly affect serum levels of YKL-40 in COPD patients.

### Positive correlation between serum level of YKL-40 and LAA% in COPD patients

**Figure 5:**
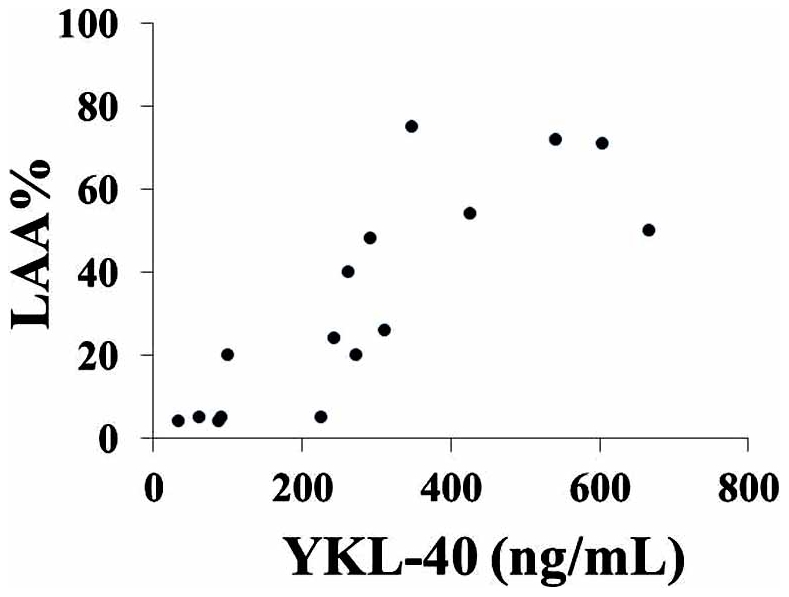
Positive correlation between serum level of YKL-40 and LAA% in COPD patients. Sixteen COPD patients had undergone high resolution computed tomography (HRCT) examination. Emphysema was assessed by using a density mask with a cutoff of −950 Hounsfield units to calculate the low-attenuation area percentage (LAA%). Serum levels of YKL-40 and LAA% were evaluated in COPD patients.

Sixteen COPD patients had undergone HRCT examination, LAA% was calculated, and serum levels of YKL-40 were examined. In COPD patients, there was a significant (P<0.0001) positive correlation between serum level of YKL-40 and LAA% (%FEV_1_) (r = 0.830) (Fig. 5).

## Discussion

Smoking is recognized to be the largest risk factor for COPD. Cigarette smoke is a major source of reactive oxygen species (ROS), exposure to which can lead to pulmonary inflammation and emphysema [2]. In fact, treatment with antioxidants has been shown to decrease the degree of oxidative damage in COPD patients and COPD animal models. For example, orally administered N-acetylcysteine (NAC) reduces the viscosity and purulence of phlegm in COPD patients [35]. CuZn superoxide dismutase is known to be a strong antioxidant, and Tg mice overproducing human CuZn superoxide dismutase do not develop pulmonary inflammation in models of pulmonary emphysema induced by cigarette smoke or elastase [36]. In addition, the antioxidant thioredoxin 1 (TRX1) inhibits elastase-induced emphysema in mice [37]. In the present study, we showed that expression of antioxidant genes such as GST, SOD1, and SOD3 was decreased in the lungs of IL-18 Tg mice. These results suggest that decreased antioxidant activities in the lungs may contribute to pulmonary inflammation and emphysema in the COPD mouse model .

Cathepsins are lysosomal cysteine proteases involved in the pathogenesis of COPD [5], [38]. Expression of cathepsin S is induced by IFN-γ in several cell types, including smooth muscle cells. Increased levels of cathepsin L have been observed in BALF of patients with emphysema, and alveolar macrophages from COPD patient secrete more cysteine protease than macrophages from smokers without disease, or those from non-smokers [5]. Overexpression of IFN-γ in the lungs induces emphysema in mice with increased expression of cathepsins B, D, H, L and S [39]. In the present study, we found that cathepsins S, D, B, Z, L and C were strongly expressed in the lungs of IL-18 Tg mice, and that this was associated with severe emphysematous changes. These results suggest that in Tg mice, overexpression of IL-18 may increase the levels of cathepsins, which may in turn induce the development of emphysematous changes in the lungs.

In the present study we found that the levels of mRNA and/or protein for the chitinase-related genes Chi3l1, Chi3l3, and AMCase were strongly increased in the lungs of IL-18 Tg mice, relative to Tg negative littermate mice, suggesting that IL-18 induces the expression of chitinase-related genes *in vivo*. Previous studies have demonstrated that IL-13 directly induces the expression of Chi3l1 *in vivo* [34] and Chi3l1 induction by cigarette smoke was found to be partly dependent on the IL-18 pathway. In contrast, IL-18 induction was not significantly modulated in the absence of the Chi3l1 gene, suggesting that Chi3l1 operates downstream of IL-18 [27]. A previous study reported that AMCase was greatly induced in lung-specific IL-13 transgenic mice over-expressing mouse IL-13 proteins in the lungs. In contrast, AMCase was not up-regulated relative to WT mice in the IL-13 (−/−) mouse asthma model [25]. These results suggest that Chi3l3 and AMCase are IL-13-driven chitinase-like proteins. We have reported that IL-18 induces both Th1 and Th2 cytokines, including IL-13 and IFN-γ *in vivo* and *in vitro* (9–11). Moreover, disruption of the IL-13 gene but not the IFN-γ gene prevented emphysema and pulmonary inflammation in SPC-IL-18 Tg mice [17]. Therefore, we hypothesized that the expression of Chi3l1 induced by IL-18 is at least partly dependent on the IL-13 pathway *in vivo*. We established IL-13 (−/−) SPC-IL-18 Tg mice by backcrossing B6 SPC-IL-18 Tg mice with B6 IL-13 (−/−) mice. However, IL-13 gene deletion did not significantly reduce the protein level of Chi3l1 in the lungs of IL-18 transgenic mice, suggesting that IL-18 drives the expression of Chi3l1 independently of the IL-13 pathway. Previous studies have demonstrated that the proinflammatory cytokines TNF-α and IL-1β both regulate the expression of YKL-40 in articular chondrocytes via NF-κB signaling [40]. Various inflammatory cytokines including IL-13, IFN-γ, IL-1α, IL-1β, and IL-12 were greatly up-regulated in the lungs of lung-specific IL-18-Tg mice [17]. Therefore it is suggested that IL-18 regulates the expression of multiple inflammatory cytokines, together with Chi3l1.

In this study, we found that Chi3l1 protein was induced in the lung of SPC-IL-18 Tg mice. However, as we examined levels of Chi3l1 protein in the lung homogenate samples, it is unclear which type of cells are the major source of Chi3l1 in these mice. Unfortunately we have not been able to identify the cellular source as the commercial anti-goat mouse Chi3l1 polyclonal antibody was not suitable for immunohistochemistry. Next, we examined whether IL-18 can induce Chi3l1 protein from lymphocytes. Spleen cells were isolated from wild type B6 mice, and were passed through nylon wool column. The nylon wool column-passed cells (2×10^6^/mL) were stimulated with recombinant human IL-2 (100 U/mL), recombinant mouse IL-18 (100 ng/mL), or IL-2 plus IL-18. Cells were stimulated for 18 hrs, and the supernatants were harvested. ELISA analysis showed that stimulation with IL-2 plus IL-18 induced IL-13 and IFN-γ production from the cells as we previously reported [9]. In contrast, stimulation with IL-2, IL-18 or IL-2 plus IL-18 did not induced Chil1 production from the spleen cells (data not shown). Our results suggest that the Chi3l1 is induced via paracrine mechanisms. Further analysis and better biochemical tools are needed to clarify these issues.

We believe that our mouse model is a useful system to help us understand the changes taking place in the lungs of smokers and those patients with COPD. Letuve *el. al*. analyzed the serum levels of YKL-40 in 15 non-smokers, 20 current smokers and 30 COPD patients (comprising 14 current smokers and 16 ex-smokers) [26]. Very recently, Matsuura conducted similar measurements in 12 non-smokers, 11 (presumably current) smokers without COPD and 18 (presumably current) smokers with COPD [27]; both studies indicated that the serum levels of YKL-40 in smokers with COPD were significantly higher than those in smokers without COPD, or in non-smokers. Matsuura also has reported that the number of YKL-40+ cells in the lungs was significantly increased in current smokers than seen in ex-smokers or nonsmokers, but serum levels of YKL-40 was not increased in current smokers [27]. It is of note that two of the previous studies [26] [27] evaluated the serum levels of YKL-40 in relatively small numbers of subjects. In the present study, we analyzed the serum levels of YKL-40 in 30 nonsmokers, 28 current smokers without COPD and 45 COPD patients (comprising 23 current smokers and 22 ex-smokers). We found that the serum levels of YKL-40 in current smokers without COPD, and those in COPD patients, were significantly higher than in non-smokers. However, the serum levels of YKL-40 did not differ significantly between current smokers with COPD and current smokers without COPD patients, and there were no significant differences in serum YKL-40 levels between current smokers with COPD and ex-smokers with COPD. Similar to our results, Agapov showed that the serum levels of YKL-40 in current- and ex-smokers with COPD were not significantly higher than those in current smokers without COPD [41]. Overall, the results suggest that cigarette smoke may increase the serum level of YKL-40.

In this study, we found there was a significant negative correlation between serum levels of YKL-40 and %FEV_1_ in COPD patients. Previous studies reported that the decline of %FEV_1_ was correlated with emphysematous changes in COPD patients [32] [33]. These results suggest that serum levels of YKL-40 are associated with emphysematous changes in COPD patients. Therefore, we evaluated the relationship of serum levels of YKL-40 with LAA% in COPD patients. We found a very close association between serum levels of YKL-40 and LAA% in COPD patients. However, there was no significant association between serum levels of IL-18 and LAA% in COPD patients (data not shown). These results suggest that YKL-40 may be involved in the development of emphysematous changes in COPD patients.

In conclusion, our results demonstrate that chitinase-related genes (including Chi3l1, Chi3l3, and AMCase) and IL-18 may play an important role in the establishment of pulmonary inflammation and emphysematous change in COPD. The lung-specific IL-18 Tg mouse is a new model that closely resembles human COPD, and thus may be useful for screening drugs that could inhibit or slow the progression of this disease.

## Supporting Information

Table S1 **In group 1, expression levels were constitutively enhanced in lungs of Tg mice more than 2-folds compared to control WT mice at 5, 9, and 13 week of age.**
(DOC)

Table S2 **In group 2, expression levels were enhanced in lungs of Tg mice more than 2 fold compared to control WT mice at 13 week of age.**
(DOC)

Table S3 **In group 3, expression levels were decreased in lungs of Tg mice more than 2-folds compared to control WT mice at 13 week of age.**
(DOC)

Table S4 **In group 4, expression levels were temporally decreased in lungs of Tg mice more than 2-folds compared to control WT mice at 9 week of age.**
(DOC)
