# Supplementary material for: Overexpression of Chitinase 3-Like 1/YKL-40 in Lung-Specific IL-18-Transgenic Mice, Smokers and COPD
Source: PLoS One. 2011 Sep 7;6(9):e24177. doi: 10.1371/journal.pone.0024177 (PMC3168485; doi:10.1371/journal.pone.0024177)
Supplement: Table S1 — In group 1, expression levels were constitutively enhanced in lungs of Tg mice more than 2-folds compared to control WT mice at 5, 9, and 13 week of age. (DOC) [file pone.0024177.s001.doc]

**Table S1. In group 1,** **expression levels were constitutively enhanced in lungs of Tg mice more than 2-folds compared to control WT mice at 5, 9, and 13 week of age.**

| **Description** | **Gene symbol** | | **Genbank accession no.** | **Fold increased vs. WT mice (mean)** |
| --- | --- | --- | --- | --- |
| chloride channel calcium activated 3 (Clca3) | clca3 | | NM_017474 | 19.74 |
| **chitinase 3-like 3 (Chi3l3)a** | chi3l3 | | NM_009892 | 4.09 |
| intelectin (Itln) | itlna | | NM_010584 | 7.33 |
| **interleukin 18 (Il18) (Transgene)a** | il18 | | NM_008360 | 5.93 |
| NEUTROPHIL GELATINASE-ASSOCIATED LIPOCALIN PRECURSOR (NGAL) (P25) | lcn2 | | BC020275 | 5.46 |
| fructose bisphosphatase 1 (Fbp1) | fbp1 | | NM_019395 | 7.87 |
| small inducible cytokine subfamily A17 (Scya17) | ccl17 | | NM_011332 | 4.03 |
| interferon-inducible GTPase (Iigp-pending) | iigp1 | | NM_021792 | 2.71 |
| solute carrier family 26, member 4 (Slc26a4) | slc26a4 | | NM_011867 | 8.21 |
| C-type (calcium dependent, carbohydrate recognition domain) lectin, superfamily member 12 (Clecsf12) | clecsf12 | | NM_020008 | 5.45 |
| polymeric immunoglobulin receptor (Pigr) | pigr | | NM_011082 | 4.93 |
| IG MU CHAIN C REGION | igh-vj558 | | BY426609 | 4.25 |
| IG MU CHAIN C REGION | igh-vj558 | | BY426609 | 8.88 |
| spi2 proteinase inhibitor (spi2/eb1) mRNA, 3 end | serpina3g | | M64085 | 4.44 |
| serum amyloid A 3 (Saa3) | saa3 | | NM_011315 | 6.10 |
| similar to IGG FC BINDING PROTEIN (FRAGMENT) [Homo sapiens] | a430096b05rik | | AK040441 | 3.49 |
| interferon gamma induced GTPase | igtp | | AK088315 | 2.01 |
| small inducible cytokine A8 (Scya8) | ccl8 | | NM_021443 | 5.31 |
| histocompatibility 2, complement component factor B (H2-Bf) | h2-bf | | NM_008198 | 3.28 |
| complement component C3 mRNA, alpha and beta subunits | c3 | K02782 | | 2.58 |
| complement component 1, q subcomponent, alpha polypeptide (C1qa) | c1qa | NM_007572 | | 3.00 |
| histocompatibility 2, class II, locus Mb1 (H2-DMb1) | h2-dmb1 | NM_010387 | | 2.54 |
| RIKEN cDNA 9230117N10 gene (9230117N10Rik) | 9230117n10rik | NM_133775 | | 3.57 |
| RIKEN cDNA 1100001H23 gene (1100001H23Rik) | 1100001h23rik | NM_025806 | | 2.36 |
| B-cell leukemia/lymphoma 2 related protein A1b (Bcl2a1b) | bcl2a1b | NM_007534 | | 4.24 |
| small inducible cytokine B subfamily (Cys-X-Cys), member 10 (Scyb10) | cxcl10 | NM_021274 | | 2.11 |
| glutamate-cysteine ligase, catalytic subunit, clone MGC:30487 IMAGE:4195425 | gclc | BC019374 | | 2.14 |
| IG MU CHAIN C REGION | igh-vj558 | BY426609 | | 7.64 |
| CD14 antigen (Cd14) | cd14 | NM_009841 | | 2.84 |
| small inducible cytokine A6 (Scya6) | ccl6 | NM_009139 | | 3.33 |
| **chitinase, acidic (AMCase)a** | 2200003e03rik | NM_023186 | | 3.69 |
| **similar to chitinase 3-like 1 (cartilage glycoprotein-39), clone MGC:7884 IMAGE:3582304 a** | chi3l1 | BC005611 | | 2.07 |
| histocompatibility 2, class II antigen A, beta 1 (H2-Ab1) | h2-ab1 | NM_010379 | | 2.06 |
| leucine-rich alpha-2-glycoprotein (Lrg-pending) | lrg1 | NM_029796 | | 3.04 |
| beta-2 microglobulin (B2m) | b2m | NM_009735 | | 2.09 |
| eosinophil-associated ribonuclease 11, clone MGC:41399 IMAGE:1445853 | ear11 | BC027557 | | 2.61 |

a. IL-18 gene and chitinase-related genes were bold.
