# Supplementary material for: Overexpression of Chitinase 3-Like 1/YKL-40 in Lung-Specific IL-18-Transgenic Mice, Smokers and COPD
Source: PLoS One. 2011 Sep 7;6(9):e24177. doi: 10.1371/journal.pone.0024177 (PMC3168485; doi:10.1371/journal.pone.0024177)
Supplement: Table S2 — In group 2, expression levels were enhanced in lungs of Tg mice more than 2 fold compared to control WT mice at 13 week of age. (DOC) [file pone.0024177.s002.doc]

**Table S2. In group 2, expression levels were enhanced in lungs of Tg mice more than 2 fold compared to control WT mice at 13 week of age.**

| **Description** | **Gene symbol** | **Genbank accession no.** | **Fold increased vs. WT mice (mean)** |
| --- | --- | --- | --- |
| IG GAMMA-1 CHAIN C REGION, MEMBRANE-BOUND FORM | Igg | ENSMUST00000003869 | 8.64 |
| cholesterol 25-hydroxylase (Ch25h) | ch25h | NM_009890 | 6.63 |
| arginase 1, liver (Arg1) | arg1 | NM_007482 | 4.81 |
| secreted phosphoprotein 1 (Spp1) | spp1 | NM_009263 | 6.29 |
| unknown EST | 1100001g20rik | AK003352 | 5.17 |
| cathepsin S (Ctss) | ctss | NM_021281 | 4.96 |
| FXYD domain-containing ion transport regulator 4 (Fxyd4) | fxyd4 | NM_033648 | 4.84 |
| triggering receptor expressed on myeloid cells 2a (Trem2a-pending) | trem2 | NM_031253 | 4.38 |
| glycoprotein (transmembrane) nmb (Gpnmb) | gpnmb | NM_053110 | 4.01 |
| glycoprotein 49 B (Gp49b) | lilrb4 | NM_013532 | 4.47 |
| IMMUNOGLOBULIN J CHAIN PRECURSOR homolog [Mus musculus] | igj | NM_152839 | 3.88 |
| cathepsin D (Ctsd) | ctsd | NM_009983 | 3.58 |
| acid phosphatase 5, tartrate resistant (Acp5) | acp5 | NM_007388 | 4.18 |
| recombinant antineuraminidase single chain Ig VH and VL domains (LOC56304) | loc56304 | NM_019633 | 3.20 |
| phospholipase D3 (Pld3) | pld3 | NM_011116 | 3.85 |
| oxidized low density lipoprotein (lectin-like) receptor 1 (Olr1) | olr1 | NM_138648 | 3.58 |
| Ig C.C58 M75 kappa light chain (VK Ser-group) | igk-v8 | X02816 | 3.31 |
