# Supplementary material for: Overexpression of Chitinase 3-Like 1/YKL-40 in Lung-Specific IL-18-Transgenic Mice, Smokers and COPD
Source: PLoS One. 2011 Sep 7;6(9):e24177. doi: 10.1371/journal.pone.0024177 (PMC3168485; doi:10.1371/journal.pone.0024177)
Supplement: Table S3 — In group 3, expression levels were decreased in lungs of Tg mice more than 2-folds compared to control WT mice at 13 week of age. (DOC) [file pone.0024177.s003.doc]

**Table S3. In group 3, expression levels were decreased in lungs of Tg mice more than 2-folds compared to control WT mice at 13 week of age.**

| **Description** | **Gene symbol** | **Genbank accession no.** | **Fold increased vs. WT mice (mean)** |
| --- | --- | --- | --- |
| spermine binding protein | sbp | AK009627 | 0.90 |
| brain expressed X-linked 2 (Bex2) | bex2 | NM_009749 | 0.40 |
| spermine binding protein (Sbp) | sbp | NM_011321 | 0.79 |
| cytochrome P450, subfamily IV B, polypeptide 1 (Cyp4b1) | cyp4b1 | NM_007823 | 0.47 |
| flavin containing monooxygenase 3 (Fmo3) | fmo3 | NM_008030 | 0.49 |
| glutathione S-transferase, alpha 3 (Gsta3) | gsta3 | NM_010356 | 0.52 |
| cytochrome P450, 2f2 (Cyp2f2) | cyp2f2 | NM_007817 | 0.32 |
| carboxylesterase 3 (Ces3) | ces3 | NM_053200 | 0.59 |
| RIKEN cDNA 2310051B21 gene (2310051B21Rik) | lipf | NM_026334 | 1.06 |
| paraoxonase 1 (Pon1) | pon1 | NM_011134 | 0.47 |
| ALPHA 8 INTEGRIN (FRAGMENT) homolog [Rattus norvegicus] | itga8 | AK031326 | 0.66 |
| cytochrome P450, 2a4 (Cyp2a4) | cyp2a4 | NM_009997 | 0.43 |
| serum deprivation response (Sdpr) | sdpr | NM_138741 | 0.59 |
| glutathione S-transferase, mu 1 (Gstm1) | gstm1 | NM_010358 | 0.46 |
| aldehyde dehydrogenase family 1, subfamily A1 (Aldh1a1) | aldh1a1 | NM_013467 | 0.56 |
| olfactory receptor MOR105-5P (MOR105-5P) | olfr430; mor105-5p | NM_146718 | 0.40 |
| PROCOLLAGEN C-TERMINAL PROTEINASE ENHANCER PROTEIN 2 | pcolce2 | AK010249 | 0.63 |
| aldehyde dehydrogenase family 1, subfamily A7 (Aldh1a7) | aldh1a7 | NM_011921 | 0.68 |
| CHLORIDE INTRACELLULAR CHANNEL 5 homolog [Rattus norvegicus] | clic5 | AK046522 | 0.64 |
| MAD homolog 7 (Drosophila) (Madh7) | smad7 | NM_008543 | 0.73 |
