# Supplementary material for: Overexpression of Chitinase 3-Like 1/YKL-40 in Lung-Specific IL-18-Transgenic Mice, Smokers and COPD
Source: PLoS One. 2011 Sep 7;6(9):e24177. doi: 10.1371/journal.pone.0024177 (PMC3168485; doi:10.1371/journal.pone.0024177)
Supplement: Table S4 — In group 4, expression levels were temporally decreased in lungs of Tg mice more than 2-folds compared to control WT mice at 9 week of age. (DOC) [file pone.0024177.s004.doc]

**Table S4. In group 4, expression levels were temporally decreased in lungs of Tg mice more than 2-folds compared to control WT mice at 9 week of age.**

| **Description** | **Gene symbol** | **Genbank accession no.** | **Fold increased vs. WT mice (mean)** |
| --- | --- | --- | --- |
| olfactory receptor MOR177-1 (MOR177-1) | olfr1132; mor177-1 | NM_146836 | 0.55 |
| parathyroid hormone (Pth) | pth | NM_020623 | 0.61 |
| clone IMAGE: 4161424 | ptprf | BC039767 | 0.75 |
| MAL2 | mal2 | AK035986 | 0.47 |
| erythrocyte protein band 4.9 (Epb4.9) | epb4.9 | NM_013514 | 0.69 |
| ATP synthase, H+ transporting, mitochondrial F1 complex, O subunit (Atp5o) | atp5o | NM_138597 | 0.90 |
| olfactory receptor MOR119-1 (MOR119-1) | olfr214; mor119-1 | NM_146759 | 0.78 |
| mJTB | jtb | AB016490 | 0.76 |
| olfactory receptor 17 (Olfr17) | olfr17 | NM_020598 | 0.78 |
| SOX15 (Sox15) | sox15 | AF182945 | 0.67 |
| similar to hypothetical protein, clone MGC:11901 IMAGE:3598565 | aa792894 | BC005704 | 0.80 |
| secreted acidic cysteine rich glycoprotein (Sparc) | sparc | NM_009242 | 0.63 |
| similar to CYTOCHROME P450 26A2 (EC 1.14.-.-) (P450RAI-2) (RETINOIC-ACID METABOLIZING CYTOCHROME) [Homo sapiens] | cyp26b1 | AK028272 | 0.49 |
| RIKEN cDNA 2400003B06 gene (2400003B06Rik) | tmed9 | NM_026211 | 1.09 |
